# Case Report: Rapid and progressive left ventricular endocardial calcification in an infant with Williams syndrome

**DOI:** 10.3389/fped.2024.1324585

**Published:** 2024-04-08

**Authors:** Jie Zhou, Dan Liu, Jiao Chen

**Affiliations:** ^1^ Department of Ultrasonic Medicine, West China Second University Hospital of Sichuan University, Chengdu, China; ^2^ Key Laboratory of Birth Defects and Related Diseases of Women and Children, Ministry of Education, Sichuan University, Chengdu, China; ^3^ Xizang Autonomous Region Women's and Children's Hospital, West China Second University Hospital of Sichuan University, Lhasa, China

**Keywords:** Williams syndrome, cardiovascular disease, endocardial calcification, arterial stenosis, infant

## Abstract

Williams syndrome (WS) is characterized by a range of clinical features, including cardiovascular disease, distinctive facial traits, neurobehavioral disorders, and a condition known as transient infantile hypercalcemia. Among these, endocardial calcification represents a non-specific response to severe, etiologically diverse myocardial injuries. In this report, we document a unique case involving an infant with WS who exhibited rapidly progressive arterial stenosis and left ventricular endocardial calcification, associated with a novel heterozygous deletion. While arterial stenosis is the most frequently observed cardiovascular issue in WS, instances of endocardial calcification during infancy are exceedingly rare and have not previously been reported in the context of WS.

## 1 Introduction

Williams syndrome (WS) is a distinctive multisystem disorder that affects the connective tissues and the cardiovascular and central nervous systems, and it is commonly associated with behavioral, developmental, and cardiovascular abnormalities. WS is caused by a *de novo* deletion of 1.5–1.8 Mb of 26–28 genes located on chromosome 7 at position 7q11.23 (1), with a prevalence rate of 1 in 8,000–20,000 newborns (2, 3). Although cardiovascular defects occur in approximately 80% of patients with WS (4), only 33% of diagnosed cardiovascular defects are detected prenatally, and they tend to be mild, so they are often ignored before birth (5). Structural cardiovascular abnormalities are present in up to 93% of patients with WS and present within the first year of life, with peripheral pulmonary artery stenosis and supravalvular aortic stenosis (SVAS) being the most common (6).

Endocardial calcification is a non-specific reaction to severe etiologically heterogeneous myocardial injuries (7). The etiology of endocardial calcification can be classified into two categories: dystrophic and metastatic, with dystrophic calcification being the main cause. Endocardial calcification may be related to hypoxic-ischemic injury; trauma, such as cardiac surgery; myocarditis (usually fungal or viral); or toxic damage due to alcohol or drugs consumption. Metastatic calcification may be present in patients with hyperparathyroidism, chronic renal failure, oxaluria and aluminum intoxication, dietary calcium and/or vitamin D deficiency, and sarcoidosis (7).

Endocardial calcification is uncommon during infancy. In this report, we present a case of a child with Williams syndrome (WS) who exhibited a typical cardiac structural defect along with rapid and progressive endocardial calcification within the left ventricle (LV).

## 2 Case presentation

A newborn baby was admitted to the neonatal department due to severe pneumonia, and she was noted to have a murmur. The baby was born to a 28-year-old G1P0 mother after elective induction at 36 + 1 weeks of gestation due to fetal distress. The neonatal birth weight was 2,100 g (p10), and the length was 44 cm (p15). The prenatal examination showed no abnormalities, except for intrauterine growth retardation. The family history was non-contributory, and the mother was not exposed to radiation or toxic substances during pregnancy. An echocardiogram at 15 days (Table 1) revealed mild-to-moderate pulmonary artery stenosis (Vmax = 3.0–3.5 m/s), and extremely mild aortic arch stenosis with a gradient of 20 mmHg with normal LV function (ejection fraction [EF] = 62%, lateral E/e′ [E/e′ Lat] = 7.8).

**Table 1:** The diameter of the affected arteries at different ages in a patient with Williams syndrome measured by echocardiography.

Affected arteries	Corresponding arterial diameter at different months of age(z score)		
	15 day	2.5 month	4 month
Main pulmonary artery	5.0 mm (−3.843)	5.0 mm (−5.591)	7.7 mm (−1.924)
Right pulmonary artery	3.0 mm (−1.091)	3.5 mm (−1.711)	3.6 mm (−1.849)
Left pulmonary artery	3.0 mm (−0.95)	3.0 mm (−3.232)	3.2 mm (−3.084)
Aortic annulus	6.8 mm (0.64)	6.3 mm (−1.332)	8.2 mm (0.903)
Aortic sinuses	7.5 mm (−1.718)	6.6 mm (−4.206)	8.3 mm (−2.315)
Aortic sinotubular junction	6.5 mm (−1.224)	3.9 mm (−6.882)	5.6 mm (−3.638)
Ascending aorta	6.5 mm (−1.224)	6.4 mm (−1.669)	7.1 mm (−1.140)
Transverse aorta	4.2 mm (−3.513)	4.5 mm (−3.998)	4.6 mm (−4.343)
Aortic isthmus	3.5 mm (−3.638)	3.9 mm (−3.787)	4.1 mm (−3.876)

**Figure 1:**
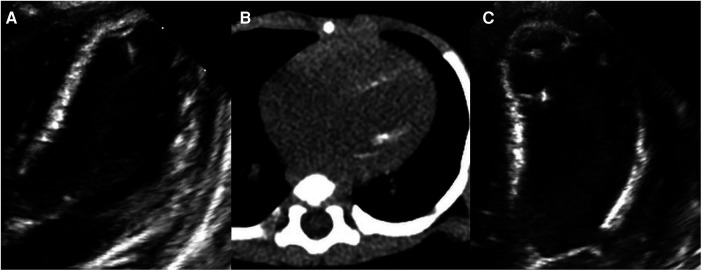
Left ventricular (LV) calcification detected by echocardiography and computed tomography angiography (CTA). At 2.5 months of age, (**A**) echocardiography showing increased echo of LV endocardium and (**B**) CTA showing diffusely distributed calcification of the LV endocardium. At 4 months of age, (**C**) echocardiography showing diffusely distributed calcification of the LV endocardium, chordae tendineae, and papillary muscle.

**Figure 2:**
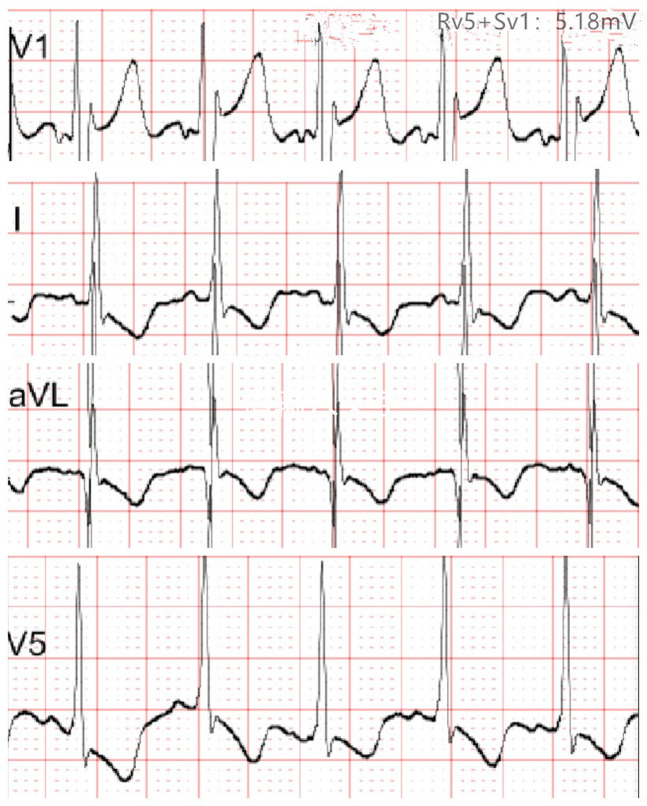
Electrocardiogram at 2.5 months of age showing T-wave flattening or inversion in leads I, aVL, and V5, and left ventricular hypertrophy was suspected.

At 2.5 months of age, the patient was admitted to our hospital due to difficulty with feeding for more than 2 months, which was initially thought to be a result of her severe reflux. The echocardiogram (Table 1) showed moderate-to-severe stenosis of the main pulmonary artery and its branches (Vmax = 3.3–4.2 m/s), a gradient of 55 mmHg across the upper aortic valve, mild aortic arch stenosis with a gradient of 36 mmHg in the setting of LV dysfunction (EF = 35%, E/e′ Lat = 16.2), and increased echo of LV endocardium (Figure 1A). The coronary arteries appeared normal. Computed tomography angiography showed aortic stenosis, pulmonary artery stenosis, and diffusely distributed calcification of the LV endocardium (Figure 1B). The patient's eyelids were swollen, and blood pressure was normal (92/32 mmHg). The electrocardiogram (Figure 2) showed ST-T changes, and LV hypertrophy was suspected. Biochemical examination (Table 2) showed myocardial injury; subclinical hypothyroidism; TORCH complex infection; normal hepatic and renal function; normal calcium (2.65 mmol/L), parathyroid hormone, and vitamin D; and no abnormalities in allergen food groups, respiratory function, and autoantibody tests. WS was highly suspected based on these findings.

**Table 2:** Biochemical examination indicators at 2.5 months of age in a patient with Williams syndrome.

Biochemical examination	Results
Myocardial Enzymes	Creatine kinase isoenzyme (CK-MB) 6.04 ug/L^a^
	Phosphocreatine kinase (CK) 115 U/L
	Troponin I (cTnI) 0.259 ug/L^a^
	Myoglobin (Myo) 73.0 ug/L
Brain natriuretic peptide	737.56 pg/ml
Thyroid function	Triiodothyronine (T3) 1.83 nmol/L
	Tetraiodothyronine (T4) 82.60 nmol/L
	Thyroid-stimulating hormone (TSH) 31.558 mIU/L^a^
	Free triiodothyronine (FT3) 5.94 pmol/L
	Free thyroid hormone (FT4) 14.01 pmol/L
The calcium level	2.65 mmol/L
Parathyroid hormone level	15.20 pg/ml
The vitamin D level	41.10 pg/ml
Allergen food groups	Normal
Allergen respiratory function	Normal
Allergen autoantibody tests	Normal
TORCH	Toxoplasma immunoglobin M (Toxo-IgM) < 3.0 AU/ml
	Toxoplasma immunoglobin G (Toxo-IgG)** **< 3.0 IU/ml
	Cytomegalovirus immunoglobin M (CMV-IgM) < 5.0 U/ml
	Cytomegalovirus immunoglobin G (CMV-IgG) 60.4 U/ml^a^
	Rubella virus immunoglobin M (Ru-IgM) < 10 AU/ml
	Rubella virus immunoglobin G (Ru-IgG) 12.2 IU/ml^a^
	Shingles virus immunoglobin M (HSV-IgM) < 5.0 Index
	Shingles virus immunoglobin G (HSV-IgG) 9.8 Index^a^

^a^ Represents abnormal results.

Whole-exome sequencing of peripheral blood from the baby and her parents revealed a new pathogenic copy number variation (1.431Mb deletion) in the 7q11.23 region (Chr7: 73303398–74733981), which contains *ELN*, confirming the diagnosis of WS. The baby was given symptomatic treatment, including prednisolone acetate, digoxin as a cardiotonic glycoside, and captopril for diuresis. Echocardiography at the age of 4 months showed improved LV systolic function (EF = 42%, E/e′ Lat = 15.9) with diffusely distributed calcification of the LV endocardium, chordae tendineae, and papillary muscle (Figure 1C). Main pulmonary artery stenosis and SVAS had improved in lesion severity, while peripheral pulmonary artery stenosis and coarctation were unchanged (Table 1).

## 3 Discussion

WS is a rare disease that can affect multiple systems, with cardiovascular defects being the most common cause of death (8). Cardiovascular abnormalities are the direct result of *ELN* deletion (5). In patients with WS, the decrease in arterial elastin content and pathological alignment of elastin fibers lead to smooth muscle overgrowth, multilayer thickening of the media of large arteries, and development of obstructive hyperplastic intimal lesions, leading to vascular diseases in the middle- and large-sized arteries. The most notable stenoses are SVAS and peripheral pulmonary artery stenosis; however, the aortic arch, descending aorta, coronary artery, renal artery, mesenteric artery, and intracranial artery can also be affected (9). Other cardiovascular defects, including tetralogy of Fallot, complete atrioventricular septal defect, total anomalous pulmonary venous return, double-chambered right ventricular, and Ebstein anomaly of the tricuspid valve, have also been reported (10). In the present case, the patient had *ELN* deletion and typical pulmonary artery stenosis, SVAS, and aortic arch stenosis.

Although the typical defect of arterial stenosis and related clinical tests led us to quickly make a diagnosis of WS, which was confirmed by whole-exome sequencing, the etiology of the infant's rapidly progressive endocardial calcification was still unclear. Endocardial calcification is very rare in infancy. At present, only one case of giant cell myocarditis and endomyocardial calcification in a 2.5-month-old infant has been reported, which was triggered by excessive maternal alcohol abuse (11). The patient had poor cardiac function, and electrocardiogram indicated myocardial ischemia. Although the combination of high end-diastolic pressure due to severe SVAS coupled with coronary artery stenosis can result in decreased coronary artery perfusion pressure and myocardial ischemia (12), ultimately resulting in cardiac dysfunction and papillary muscle and endocardial calcification, it often takes a long time to develop. In the present study, echocardiography showed no significant coronary artery stenosis, so the decrease in cardiac function and endocardial calcification could not be explained by SVAS alone. In the present case, calcification of the endocardium progressed rapidly, and biochemical tests excluded calcium deposition, hyperparathyroidism, vitamin D deficiency, renal failure, allergy, and autoantibodies as the cause. The infant's myocardial enzymes were elevated. The TORCH complex examination showed that cytomegalovirus (CMV), rubella virus, and shingles virus immunoglobin G antibodies were positive. The infant had not been vaccinated, indicating that she was previously infected. Dystrophic cardiac calcification is related to CMV infection, and some scholars have found that CMV-infected mice have localized cardiac calcification, mainly in the right ventricle (13). Other animal experiments (14) have shown that mice with *ELN* deletion develop cardiac phenotypes attributed to aortic stenosis, including compromised fractional shortening, cardiac enlargement, necrosis, and calcification in the cardiac chambers. Based on the above literature, although there were no other related clinical symptoms in the present case, myocardial damage caused by CMV infection combined with SVAS seemed to be the most likely cause of the rapid and progressive LV endocardial calcification in the present case, although the possibility of genetic susceptibility caused by delection of new heterozygosity cannot be ruled out.

## 4 Conclusion

We report a case of progressive arterial stenosis accompanied by endocardial calcification in an infant with Williams syndrome (WS). To our knowledge, endocardial calcification in WS has not been documented before. After ruling out other potential pathogenic causes, we believe that myocardial injury due to Cytomegalovirus (CMV) infection compounded by aortic stenosis is the probable cause. Nonetheless, we cannot overlook the possibility that a genetic predisposition due to a newly identified heterozygous deletion may have played a role.

## Data Availability

The raw data supporting the conclusions of this article will be made available by the authors, without undue reservation.
